# Association Between Gut Microbiota and CD4 Recovery in HIV-1 Infected Patients

**DOI:** 10.3389/fmicb.2018.01451

**Published:** 2018-07-02

**Authors:** Wei Lu, Yuqing Feng, Fanhui Jing, Yang Han, Na Lyu, Fei Liu, Jing Li, Xiaojing Song, Jing Xie, Zhifeng Qiu, Ting Zhu, Bertrand Routy, Jean-Pierre Routy, Taisheng Li, Baoli Zhu

**Affiliations:** ^1^Department of Infectious Disease, Peking Union Medical College Hospital, Beijing, China; ^2^Center for AIDS Research, Chinese Academy of Medical Sciences and Peking Union Medical College, Beijing, China; ^3^CAS Key Laboratory of Pathogenic Microbiology and Immunology, Institute of Microbiology, Chinese Academy of Sciences, Beijing, China; ^4^Savaid School of Medicine, University of Chinese Academy of Sciences, Beijing, China; ^5^Beijing Key Laboratory of Microbial Drug Resistance and Resistome, Beijing, China; ^6^Division of Hematology and Oncology Division, Department of Medicine, Centre Hospitalier de l’Université de Montréal (CHUM), Montreal, QC, Canada; ^7^Centre de Recherche du Centre Hospitalier de l’Université de Montréal (CRCHUM), Montreal, QC, Canada; ^8^Chronic Viral Illnesses Service Research Institute and Division of Hematology, McGill University Health Centre, Montreal, QC, Canada; ^9^Collaborative Innovation Center for Diagnosis and Treatment of Infectious Diseases, The First Affiliated Hospital, College of Medicine, Zhejiang University, Hangzhou, China; ^10^Department of Pathogenic Biology, School of Basic Medical Sciences, Southwest Medical University, Luzhou, China

**Keywords:** HIV-infected individuals, gut microbiota, metagenomics sequencing, CD4 recovery, butyrate-producing bacteria

## Abstract

Composition of the gut microbiota has been linked with human immunedeficiency virus (HIV)-infected patients on antiretroviral therapy (ART). Evidence suggests that ART-treated patients with poor CD4^+^ T-cell recovery have higher levels of microbial translocation and immune activation. However, the association of the gut microbiota and immune recovery remains unclear. We performed a cross-sectional study on 30 healthy controls (HC) and 61 HIV-infected individuals, including 15 immunological ART responders (IRs), 20 immunological ART non-responders (INRs) and 26 untreated individuals (VU). IR and INR groups were classified by CD4^+^ T-cell counts of ≥350 cells/mm^3^ and <350 cells/mm^3^ after 2 years of ART, respectively. Each subject’s gut microbiota composition was analyzed by metagenomics sequencing. Levels of CD4^+^ T cells, CD8^+^HLA-DR^+^ T cells and CD8^+^CD38^+^ T cells were measured by flow cytometry. We identified more *Prevotella* and fewer *Bacteroides* in HIV-infected individuals than in HC. Patients in INR group were enriched with *Faecalibacterium prausnitzii*, unclassified *Subdoligranulum sp.* and *Coprococcus comes* when compared with those in IR group. *F. prausnitzii* and unclassified *Subdoligranulum sp.* were overrepresented in individuals in VU group with CD4^+^ T-cell counts <350 cells/mm^3^. Moreover, we found that the relative abundance of unclassified *Subdoligranulum sp.* and *C. comes* were positively correlated with CD8^+^HLA-DR^+^ T-cell count and CD8^+^HLA-DR^+^/CD8^+^ percentage. Our study has shown that gut microbiota changes were associated with CD4^+^ T-cell counts and immune activation in HIV-infected subjects. Interventions to reverse gut dysbiosis and inhibit immune activation could be a new strategy for improving immune reconstitution of HIV-1-infected individuals.

## Introduction

The human gastrointestinal tract is a complex, dynamic ecosystem and consists of a large number of microorganisms. The gut microbiota is made of 3 × 10^13^ bacteria, archaea, viral, parasites, and fungal species (Sender et al., 2016). The gut microbiota influences many physiological processes, such as nutrient transformation and absorption, drug metabolism, development and function of immune system (Xiong et al., 2015) and has also been associated with type 2 diabetes (T2D) (Qin et al., 2012), cardiovascular disease (Koeth et al., 2013), response to cancer immunotherapy (Routy et al., 2018), as well as human immunodeficiency virus (HIV) infection (Vujkovic-Cvijin et al., 2013). Converging data from many cross-sectional studies suggest that gut microbiota shifts from *Bacteroides* to *Prevotella* predominance after HIV infection (Lozupone et al., 2013; Mutlu et al., 2014; Vázquez-Castellanos et al., 2015; Ling et al., 2016; Dillon et al., 2017; Serrano-Villar et al., 2017).

Most of combinations of antiretroviral therapy (ART)-treated patients can achieve distinct viral load reduction as well as CD4^+^ T-cell reconstitution in peripheral blood (Hammer et al., 1997) and consequently can live near-normal lifespans (Samji et al., 2013). However, the extent of immunologic recovery varies greatly between individuals and some patients can only have insufficient reconstitution of CD4^+^ T cells despite achieving virologic suppression after ART (Lu et al., 2015). These individuals are referred as immunologic non-responders (INRs) compared to those patients with robust CD4^+^ T cells recovery (Immunologic responders, IRs) (Morse and Kovacs, 2008). Residual systemic chronic immune activation persists more in INRs and contributes to CD4^+^ T-cell low recovery with a higher rate for progression to acquired immunodeficiency syndrome (AIDS) (Paiardini and Müllertrutwin, 2013). Monaco et al. (2016) demonstrated that lower peripheral CD4^+^ T-cell count recovery was associated with microbial transloation and increased abundance of *Enterobacteriaceae* in gut microbiota (Ponte et al., 2016). Furthermore, experimental administration of bacterial products, such as lipopolysaccharide (LPS), in natural hosts induces immune activation, which is in turn associated with increased viral load and CD4^+^ T-cell depletion (Brenchley et al., 2006). The increase in the number and fraction of CD8^+^ T cells is also a prominent feature of HIV-1-infected individuals. The activated CD8^+^ T cells, which express HLA-DR and CD38 antigens, are stronger indicators of AIDS and all cause mortality than either CD4^+^ T-cell count or plasma viral load (Imamichi et al., 2012).

Previous studies have revealed alterations of gut microbiota following HIV infection and ART administration (Ling et al., 2016). However, these studies were limited to the use of 16S rRNA gene amplicon sequencing, and analyzed at the level of genus. Besides, the differences of gut microbiota between the IR and INR groups have not been elucidated thus far. We therefore applied whole genome sequencing technology to profile gut microbiota in individuals from both IR and INR groups compared to matched healthy volunteers and explored the association between immune recovery down to the species level.

## Materials and Methods

### Subjects and Sample Collection

Patients with chronic HIV infection, receiving ART, having an undetectable plasma HIV RNA level for more than 2 years were recruited from the Department of Infectious Diseases, Peking Union Medical College Hospital, China from December 2015 to September 2016. They were divided into two groups depending on whether they were immunological responders (IRs, *n* = 15) or not (INRs, *n* = 20) (IRs and INRs, CD4^+^ T-cell counts ≥350 cells/mm^3^ and <350 cells/mm^3^ after 2 years of ART, respectively). A total of 26 treatment-naïve patients with chronic HIV-1 infection (VU) and 30 healthy matched controls were also enrolled. Ten healthy controls were from our cohort, the other 20 from our unpublished data with the same criteria in another cohort in Beijing (Zheng et al., unpublished). Subjects who have used antibiotics, probiotics, or prebiotics or have experienced diarrhea or digestive symptoms within the previous 1 month were excluded. In addition, the patients with active opportunistic infection and co-infection of HBV and HCV were also excluded from our cohort. The study received approval from the Ethics Committee of the Peking Union Medical College Hospital and the study was conducted in accordance with the approved guidelines (Ethics approval number # JS-985). Signed informed consent was obtained from each subject prior to enrolment. The T-cell subjects were determined using a FACScanto flow cytometer (BD Immunocytometry Systems) (Lozupone et al., 2013). Immunophenotyping of peripherial blood lymphocytes was analyzed by three-color flow cytometry (Epics XL flow cytometry; Beckman Coulter, United States) as previous described (Effros et al., 2003; Cooper et al., 2013). Freshly collected EDTA-anticoagulated whole blood was incubated and test with a panel of monoclonal antibodies directed against fluorescein isothiocyanate/phycoerythrin/peridinin chlorophyll protein combinations of CD3/CD4/CD8, CD3/CD16CD56/CD19, HLA-DR/CD8/CD38, and CD4/CD8/CD28 and isotype controls (Immunotech, France) (**Supplementary Figures S4–S9** and **Supplementary Table S1**). For example, the level of expression of CD28 varies, depending on the lineage and the activation state (Delves and Roitt, 1998). Approximately two grams of fresh fecal sample were placed in a collection tube in PSP^®^ Spin Stool DNA Plus Kit (Stratec co., Germany), and stored at −80°C until DNA extraction. Bead beating was used during DNA extraction to improve efficiency (Yu and Morrison, 2004).

### DNA Library Construction and Sequencing

DNA library was constructed as per the manufacturer’s instruction (Illumina, United States). In brief, one paired-end library with insert the size of 350 bp for each sample was constructed and sequenced with 150 bp read length from each end on HiSeq 2500 Illumina sequencers. The raw sequencing data was processed using the MOCAT2 (Kultima et al., 2016) pipeline to remove low-quality reads, adapters and human DNA contamination. Gut microbiota was sequenced and it generated approx. a total of 640 GB raw sequencing data for 91 fecal samples (7.0 GB per sample) was obtained (**Supplementary Table S2**). The trimmed raw reads were assembled and integrated into 1.8 million non-redundant gene catalog. The sequence data from this study are deposited in the GenBank Sequence Read Archive with the accession number SRP111623.

### Taxonomical Analysis

The taxonomic assignment and abundance estimation was performed with MetaPhlAn 2.0 (Truong et al., 2015) using default parameters. MetaPhlAn2 contains ∼1 million markers extracted from more than 7500 species. Taxonomical analysis was performed using default parameters.

### Microbial Community Types (Enterotypes)

The community types of each sample were analyzed using relative abundance of genera. The community type of each fecal metagenomic samples was analyzed with the same identification method as described in the original paper of enterotypes (Arumugam et al., 2011). Fisher’s exact test was used to calculate the significant level of the enterotype.

### PERMANOVA on the Influence of Clinical Factors

Permutational multivariate analysis of variance (PERMANOVA) was performed on the species abundance profile of all samples to assess the effect of age and subsets of T-cell on the composition of microbiota. Bray-Curtis distance and 9,999 permutations was used to obtain the permuted p-value in R [3.3.3, “vegan” package (Zapala and Schork, 2006)].

### Gene Catalog Construction

Raw DNA sequence data were *de novo* assembled using SOAPdenovo v2 (Luo et al., 2012), followed by gene prediction for high quality reads of 71 samples using MetaGeneMark v2.8 (Noguchi et al., 2006), respectively. Reference gene catalogs were clustered using CD-HIT v4.6 (Fu et al., 2012).

### Functional Annotation

All predicted genes were translated to amino acid sequences and aligned with the KEGG database using DIAMOND (Buchfink et al., 2015). Each protein was assigned to a KEGG orthologue based on the best hit gene in the KEGG database. For a certain strain, the annotation of genes was analyzed using Rapid Annotation using Subsystem Technology (RAST) to find out certain genes that related to butyrate generation (Overbeek et al., 2014).

### Pathway Analysis

Whole genome metagenomics pathway analysis was adopted in the HMP Unified Metabolic Analysis Network 2 (HUMAnN2) (Abubucker et al., 2012) pipeline to assess the potential differences in metabolic pathway. Pathway analysis was performed using default parameters. Differentially enriched pathways were identified according to their reporter score from the Z-scores of individual pathways. One-tail Wilcoxon rank-sum test was performed on all the pathways and adjusted for multiple testing using the Benjamin-Hochberg procedure (Benjamini and Hochberg, 1995). The calculation of the Z-scores was done following the formula mentioned in a previous study (Feng et al., 2015). The choices of Z-scores were dependent on the average or the median of the pathways.

### Statistical Analysis

All statistical analyses were conducted in R software. Differences between populations of demographic and clinical characteristics have been analyzed using Kruskal-Wallis test. Differential abundance of species and pathways were tested by two-tailed Wilcoxon rank-sum test. When multiple hypotheses were considered simultaneously, *p*-values were adjusted to control the false discovery rate with the method described previously (Benjamini and Hochberg, 1995). To avoid excessive correction, the cut-off value of average relative abundance was set as 10^−3^. Correlation between subsets of T cell and species were tested by Spearman’s correlation.

## Results

### Study Population

This study included 61 male subjects who had sex with men (MSM) and 30 male matched healthy controls. No differences were observed between groups in terms of body mass index (BMI), and CD8^+^ T-cell count (**Table 1**). Compared to IRs, INRs featured significantly lower CD4^+^ T-cell as per inclusion criteria, longer duration of ART, and lower CD4/CD8 ratio. Although the CD4^+^ T-cell counts in the IR group were higher than those in the INR group before the initiation of ART (*p* = 0.002). The former still experienced better recovery on the absolute number increase of CD4^+^ T-cell count compared with the INR group (*p* = 0.010).

**Table 1:** Demographic and clinical characteristics of the study population.

	VU (*n* = 26)	IR (*n* = 15)	INR (*n* = 20)	HC (*n* = 30)	*p* value
Age (years, IQR)	33.0 (27.3–39.0)	31.0 (26.0–34.5)	37.5 (34.0–44.3)	29.5 (24.0–45.8)	0.050
Male gender (No.%)	26 (100%)	15 (100%)	20 (100%)	30 (100%)	–
BMI (kg/m^2^, IQR)	20.8 (19.5–24.1)	23.2 (18.9–24.1)	22.5 (20.8–24.8)	23.3 (21.6–25.2)	0.228
ART duration (years, IQR)	–	2.7 (2.1–3.0)	3.7 (3.1–5.2)	–	0.005
CD4^+^ T-cell count (cells/mm^3^, IQR)	351.0 (182.8–423.8)	660.0 (539.5–795.0)	295.5 (214.0–352.0)	–	0.000
CD4^+^ T-cell % (IQR)	22.1 (17.5–25.3)	31.4 (27.5–38.2)	21.1 (13.0–25.6)	–	0.230
CD8^+^ T-cell count (cells/mm^3^, IQR)	648.5 (586.8–1046.0)	693.0 (623.0–862.5)	577.0 (479.5–799.0)	–	0.368
CD8^+^ T-cell % (IQR)	51.6 (42.5–56.4)	33.7 (32.1–40.9)	43.5 (37.8–50.1)	–	0.000
CD4/CD8 percentage (IQR)	0.45 (0.28–0.56)	0.94 (0.73–1.05)	0.48 (0.28–0.72)	–	0.000
Nadir CD4^+^ T-cell count (cells/mm^3^, IQR)	86.0 (44.5–158.3)	178.0 (107.5–260.0)	47.5 (34.0–72.5)	–	0.000

### Gut Microbiota Dysbiosis in HIV-Infected Individuals

**FIGURE 1:**
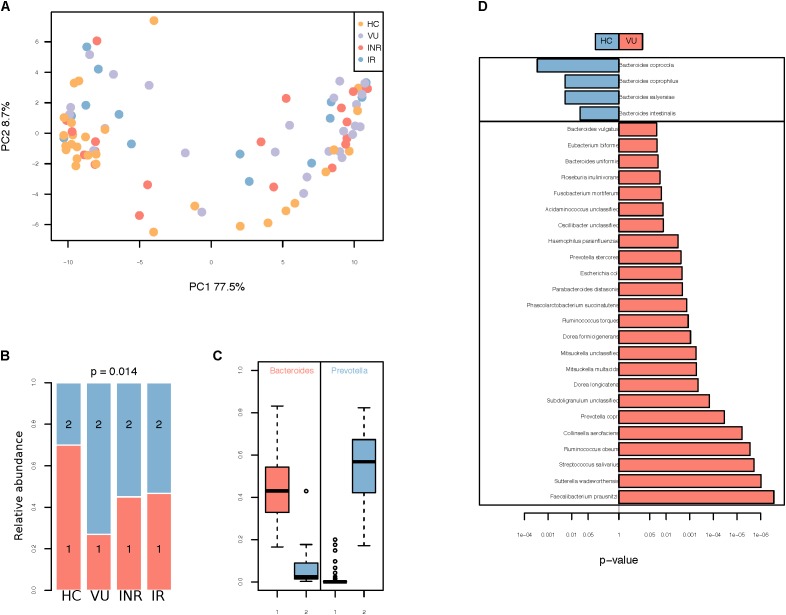
**(A)** Principal coordinates analysis (PCoA) using Bray–Curtis dissimilarity distance. **(B)** Distribution of VU, IR, INR, and HC in enterotypes. The areas of the columns scale with sample size, that is, *n* = 26, 20, 15, and 30, respectively. **(C)** Relative abundances of *Bacteroides* and *Prevotella* in the two community types. **(D)** Relative abundance of species different between VU and healthy groups. Only the median relative abundances greater than 0.1% of total abundance are included (FDR < 0.1, Wilcoxon rank-sum test corrected by the Benjamini and Hochberg method).

Phylogenetic profiling identified 428 species from collected fecal samples (**Supplementary Table S3**). There were no significant differences in alpha diversity between all the four groups (**Supplementary Figure S1A**). To identify possible differences between the bacterial components of subjects in these four group, we calculated the beta diversity of the samples using two estimators. Principal coordinate analysis (PCoA) (**Figure 1A**) and non-metric multidimensional scaling analysis revealed a clear separation of healthy controls from the other groups at the genus level (**Supplementary Figures S1B–F**). This data suggested that gut microbiota dysbiosis in HIV-infected individuals might be due to HIV infection itself other than influence of antiretroviral therapy.

Two enterotypes were identified, enterotype 1 dominated by *Bacteroides* and enterotype 2 dominated by *Prevotella* (**Figures 1B,C** and **Supplementary Table S4**). Those untreated patients had the highest percentage of enterotype 2 microbiota in gut compared with the others (*p* = 0.014, Fisher’s exact test). Individuals on ART had a lower percentage of enterotype 2 compared to VU group (*p* = 0.003, Fisher’s exact test), but it was still higher than healthy group (*p* = 0.078, Fisher’s exact test). Relative abundances of both enterotypes were similar in IR and INR groups (*p* = 1.000, Fisher’s exact test).

When comparing bacteria with relative abundance of more than 10^−3^ between VU group and HC group, we found four species were enriched in VU group and 24 species were abundant in HC group (Wilcoxon rank-sum test, FDR < 0.1, **Figure 1D** and **Supplementary Table S5**). Species more abundant in VU group mostly belong to the genus *Bacteroides* (*n* = 4), including *Bacteroides coprophilus*, *Bacteroides coprocola*, *Bacteroides intestinalis*, and *Bacteroides salyersiae*. We also observed increased *Preovtella copri* and *Prevotella stercorea* in VU group. Both of the two species belong to the genus *Prevotella*, which has been widely reported to be associated with HIV-1 infection in western countries (Lozupone et al., 2013; Mutlu et al., 2014; Vázquez-Castellanos et al., 2015; Ling et al., 2016; Dillon et al., 2017; Serrano-Villar et al., 2017).

### Association Between Gut Microbiota and CD4^+^ T-Cell Counts

**FIGURE 2:**
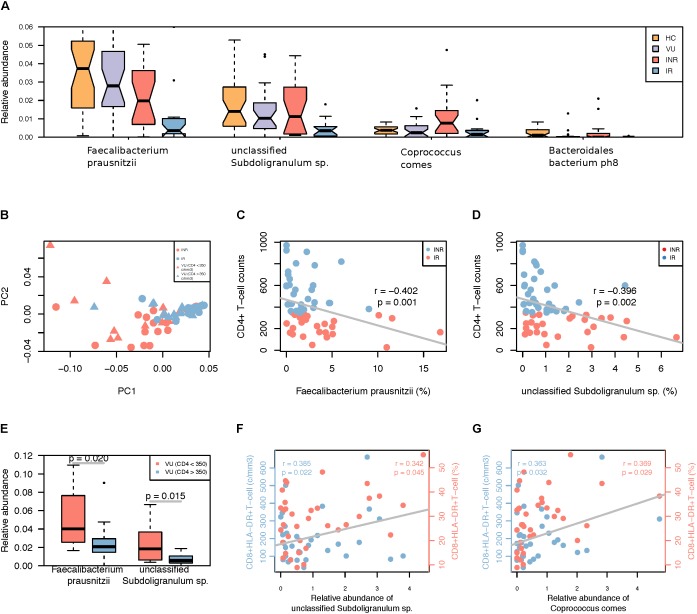
**(A)** Differentially abundant species in the IR and INR groups. Only the median relative abundances greater than 0.1% of total abundance are included (FDR < 0.3, Wilcoxon rank-sum test corrected by the Benjamini and Hochberg method). **(B)** PCoA analysis based on the relative abundance of species different between IR and INR groups. **(C)** Spearman’s correlation between *Faecalibacterium prausnitzii* and CD4^+^ T-cell counts among all HIV-1 patients. **(D)** Spearman’s correlation between unclassified *Subdoligranulum sp.* and CD4^+^ T-cell counts among all HIV-1 patients. **(E)** Relative abundance of the two species in the subgroup of VU group based on the CD4+ T-cell counts. **(F)** Spearman’s correlation between unclassified *Subdoligranulum sp.* and CD8^+^HLA-DR^+^ T-cell counts and ratio in the IR and INR groups. Blue: correlation between unclassified *Subdoligranulum sp.* and CD8^+^HLA-DR^+^ T-cell counts; Red: correlation between unclassified *Subdoligranulum sp.* and CD8^+^HLA-DR^+^ T-cell ratio. **(G)** Spearman’s correlation between *C. comes* and CD8^+^HLA-DR^+^ T-cell counts and ratio in the IR and INR groups. Blue: correlation between *C. comes* and CD8^+^HLA-DR^+^ T-cell counts; Red: correlation between *C. comes* and CD8^+^HLA-DR^+^ T-cell ratio.

A lower abundance of *F. prausnitzii* and *Haemophilus parainfluenzae* was observed in IR and INR groups compared to VU group (FDR < 0.3). When comparing gut microbiota between the IR and INR groups, four species (*F. prausnitzii*, unclassified *Subdoligranulum. sp.*, *C. comes*, and *Bacteroidales bacterium ph8*) were more abundant in the INR group (FDR < 0.3, **Figure 2A**). Interestingly, RAST showed *F. prausnitzii*, unclassified *Subdoligranulum sp.* and *C. comes* have the ability of producing butyrate.

Compared with VU group, we noticed lower abundances of *F. prausnitzii*, unclassified *Subdoligranulum sp.*, and *B. bacterium ph8* in IR group, and a higher abundance of *C. comes* in INR group. The result of PCoA analysis based on these four species of untreated patients with CD4^+^ T-cell counts >350 cells/mm^3^ was similar to that of the IR group (**Figure 2B**). Furthermore, higher CD4^+^ T-cell counts (>350 cells/mm^3^) were accompanied with lower relative abundances of *F. prausnitzii* and unclassified *Subdoligranulum sp.* (**Figures 2C,D**) but also in all HIV-1-infected patients (Wilcoxon rank-sum test, **Figure 2E**). Therefore, the abundance of *F. prausnitzii* and unclassified *Subdoligranulum sp.* might have a close association with the CD4^+^ T-cell counts independent of ART.

### Correlation Between Demographic, Clinical Data and Gut Microbiota Composition

To clarify the association between age, T-cell subsets and the composition of gut microbiota, multivariate analysis was performed by PERMANOVA. We found that age, CD4^+^ T-cell (cells/mm^3^), memory CD4^+^ T-cell (cells/mm^3^), CD4^+^CD28^+^ T-cell (%), and CD8^+^CD38^+^ T-cell (cells/mm^3^, %) were all relevant to the changes of gut microbiota among all the four groups (**Supplementary Table S6** and **Supplementary Figure S2**). And the effects of age and CD4^+^ T-cell ratio were independent. When we focused on the IR and INR groups, age was no more a confounding factors in further analysis.

### Correlation Between Gut Microbiota and T Cell Activation

The increase in the number and frequency of CD8^+^ T-cells is a prominent feature of HIV infected individuals (Cao et al., 2016). The activated CD8^+^ T-cells which express HLA-DR and CD38 antigens are better indicators of AIDS and death than either CD4^+^ T-cell count or plasma viral load (Imamichi et al., 2012). To further analyze the relationship between gut microbiota dysbiosis and T-cell activation, correlation test were performed by calculating Spearman’s correlation coefficient (rho). We found that the relative abundances of unclassified *Subdoligranulum sp.* and *C. comes* were positively correlated with CD8^+^ HLA-DR^+^ T-cell counts and percentages (**Figures 2F,G**). It might suggest the increase of these two species are closely associated with T-cell activation. However, *F. prausnitzii*, unclassified *Subdoligranulum sp.*, *Bacteroidales bacterium ph8*, and *C. comes* were not relevant to the changes of CD8^+^CD38^+^ T-cells (data not shown).

### Differences in Microbiota Metabolic Pathways Among the Groups

**FIGURE 3:**
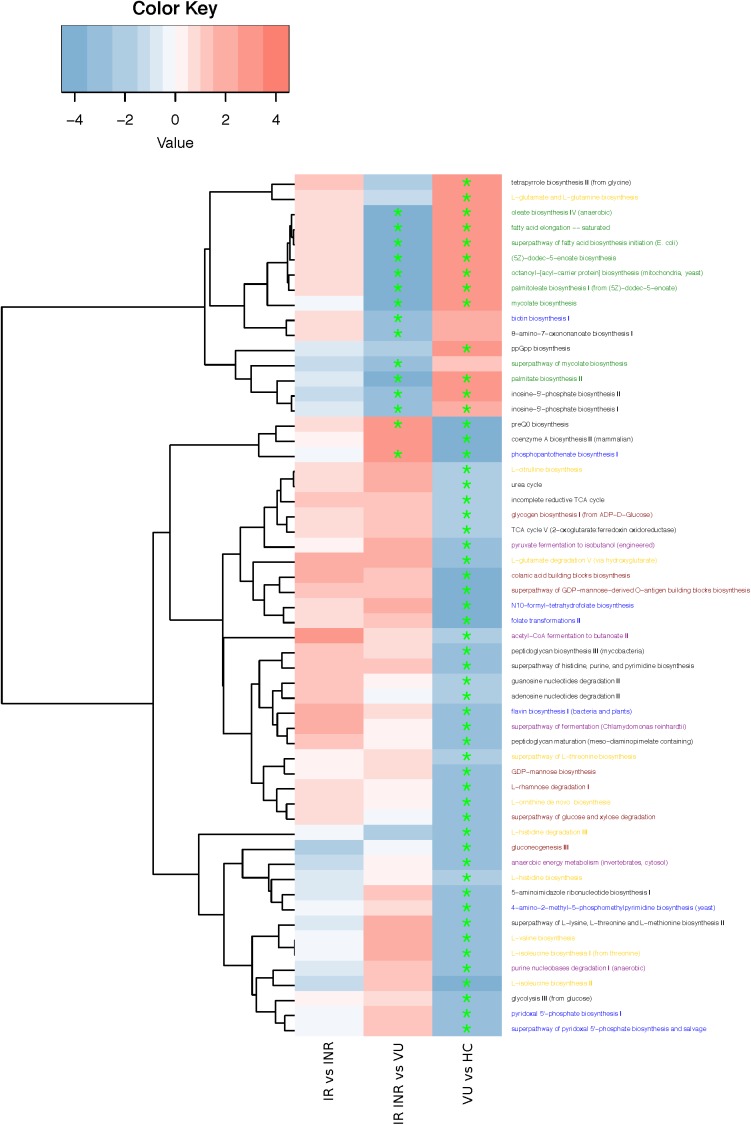
Heatmap and hierarchical clustering of pathways enriched or decreased between any of the two groups. Red: higher in the former group; blue: higher in the latter group. ^∗^FDR < 0.1. Pathway annotation marked in yellow (amino acid biosynthesis), green (fatty acid biosynthesis), blue (vitamin biosynthesis), red (carbohydrates biosynthesis), and purple (fermentation).

To investigate the functional role of gut microbiota in HIV infection, gene functions were analyzed by HUMAnN2 pipeline in our study. Pathways were mainly involved in amino acid, fatty acid, vitamin, and carbohydrates biosynthesis and fermentation (**Figure 3** and **Supplementary Table S7**). Fatty acid biosynthesis related pathways increased in HIV-infected patients, but the others all decreased compared with healthy control (FDR < 0.1). Comparison of pathways between IR/INR and VU groups showed that ART treatment could reverse the increase of fatty acid biosynthesis. It is noteworthy that pathways involved in biotin and vitamin B5 biosynthesis decreased after ART treatment. Our data showed that the relative abundance of butyrate-producing bacteria was lower and the pyruvate fermentation pathway was down regulated in the IR group compared with the INR group (*p* = 0.099 and *p* = 0.237, **Supplementary Figure S3** and **Supplementary Tables S8**,**S9**).

## Discussion

Our present study showed that in INR group was enriched with the relative abundance of *F. prausnitzii*, *Subdoligranulum sp.* and *C. comes* when compared with IR group. These species have the ability of butyrate production. Further analysis showed that they had a close connection with the CD4^+^ T-cell counts and T-cell immune activation.

Indeed, the microbiome involvement in the transmission and pathogenesis of HIV infection is being acknowledged with the changes of relative abundances of *Prevotella* and *Bacteroides* (Lozupone et al., 2013; Mutlu et al., 2014; Vázquez-Castellanos et al., 2015; Ling et al., 2016; Dillon et al., 2017; Serrano-Villar et al., 2017) (**Supplementary Table S10**). Although our study is based on Chinese population, our results still confirmed the same microbiota composition changes after HIV-1 infection previously observed in Western countries. We also confirmed that the diversity and composition of gut microbiota could not be recovered completely after effective ART which means that HIV-1 infection plays a significant role in disruption of gut homeostasis but could not restore normal gut environment of HIV-infected patients (Ponte et al., 2016).

Interestingly we found is that the relative abundance of *F. prausnitzii* and *Subdoligranulum sp.* have a close connection with CD4^+^ T-cell counts. These two species may have persistent effect on the depletion of CD4^+^ T cells after HIV infection and may lead to immune non-respondence even after effective ART. Monaco and her colleagues (Monaco et al., 2016) also showed that individuals with higher CD4^+^ T-cell counts were accompanied with lower *F. parusnitzii*, with 200 cells/mm^3^ as the cut-off value of CD4^+^ T-cell counts. These findings might disclose that gut dysbiosis has a crucial role in CD4^+^ T-cell reconstitution since the beginning of HIV infection other than any antiretroviral therapy afterward. Explanations for this association needs to be elucidated in future studies.

Systemic chronic immune activation is considered as the driving force of CD4^+^ T-cell depletion and AIDS (Wada et al., 2015). The residual immune activation may represent a therapeutic target to improve the prognosis of HIV-infected individuals receiving ART (Paiardini and Müllertrutwin, 2013). Bacterial components (LPS, peptidoglycan, and bacterial DNA) may further stimulate the vicious circle of immune activation, which in turn promotes viral replication and disease progression (Gori et al., 2011). We found a positive correlation between *Subdoligrnulum sp*., *C. comes* and CD8^+^HLA-DR^+^ T cells, indicating changes of gut microbiota may be involved in immune activation. These two species were thought to be beneficial for human health, due to the fact that they have the ability of buryrate production (Louis and Flint, 2009; Venessa et al., 2011). They might take some role in mechanism of immune recovery in HIV-1 infection.

Our study has some limitations. Firstly, it is a cross-sectional study based on a small number of Chinese subjects. Secondly, though ART could not restore HIV-associated microbial dysbiosis, it remains hard to differentiate the effect of different antitroviral regimen on gut microbiome as in the literature (Pinto-Cardoso et al., 2018; Shilaih et al., 2018). Moreover, whether or not gut microbes have impact on antiretroviral durg metabolism needs to be studied further (Klatt et al., 2017). A larger scale of longtitudinal study on naïve-treatment patients before and after different combination of ART will be helpful to answer the above questions.

In summary, our results indicate that HIV infection is the main influencing factor of gut microbiome composition. The enrichment of some butyrate-producing bacteria is associated with poor CD4^+^ T-cell reconstruction, and further studies are needed to reveal the underlying mechanism. Modifying the composition of gut microbiota, for example decreasing butyrate-producing bacteria composition, might be new strategies to enhance immune reconstitution for HIV patients.

## Availability of Data and Materials

The datasets supporting the conclusions of this article are included within the article and in additional files. R version 3.3.3 was used with packages ggplot2 version 2.2.1, ggplots version 3.0.1, grid version 3.3.3, and vegan version 2.4-3.

## Ethics Statement

This study was carried out in accordance with the recommendations of Declaration of Helsinki, the Ethics Committee of the Peking Union Medical College Hospital. The protocol was approved by the the Ethics Committee of the Peking Union Medical College Hospital. All subjects gave written informed consent in accordance with the Declaration of Helsinki.

## Author Contributions

WL, TL, and BZ designed the project. YF, YH, NL, JL, XS, JX, ZQ, and TZ did the experiments. FL did the bioinformatics analysis. YF and FJ wrote the initial manuscript. BR and J-PR provided invaluable feedback and insights into analyses and the manuscript. All authors approved the final version of the manuscript.

## Conflict of Interest Statement

The authors declare that the research was conducted in the absence of any commercial or financial relationships that could be construed as a potential conflict of interest.

## Supplementary Material

FIGURE S1 The differences of microbiota composition among the four groups. **(A)** Shannon index. **(B)** Non-metric multidimensional scaling (NMDS) of the bacterial composition at the genus level. **(C,D)** The distribution of the points along PC1 and PC2 are shown in the boxes. **(E,F)** The distribution of the points along NMDS1 and NMDS2 are shown in the boxes.

FIGURE S2 Redundancy Analysis (RDA) of the correlation between some clinical index and species in IR and INR groups. Red dot: patients from the INR group; blue dot: patients from the IR group; black dot: species; green line: clinical index.

FIGURE S3 **(A)** Relative abundance of butyrate-producing bacteria. **(B)** Relative abundance of butyrate-producing pathway, byruvate fermentation to butanoate, acquired from HUMAnN2.

FIGURE S4 Flow cytometry analysis of peripheral blood in patient-1 (part 1).

FIGURE S5 Flow cytometry analysis of peripheral blood in patient-1 (part 2).

FIGURE S6 Flow cytometry analysis of peripheral blood in patient-1 (part 3).

FIGURE S7 Flow cytometry analysis of peripheral blood in patient-2 (part 1).

FIGURE S8 Flow cytometry analysis of peripheral blood in patient-2 (part 2).

FIGURE S9 Flow cytometry analysis of peripheral blood in patient-2 (part 3).

TABLE S1 Clinical index of our cohort.

TABLE S2 Read QC Statistics.

TABLE S3 Detail information on species level analyzed by MetaPhlAn.

TABLE S4 Enterotypes detail information.

TABLE S5 Different species among the four groups.

TABLE S6 PERMANOVA analysis results.

TABLE S7 Different pathways among the four groups based on HUMAnN2.

TABLE S8 Relative abundance of butyrate-producing bacteria.

TABLE S9 Relative abundance of pyruvate fermentation to butanoate.

TABLE S10 Studies on gut microbiome associated with HIV infection and HAART treatment.
