# Supplementary figures and images for: Association Between Gut Microbiota and CD4 Recovery in HIV-1 Infected Patients
Source: Front Microbiol. 2018 Jul 2;9:1451. doi: 10.3389/fmicb.2018.01451 (PMC6043814; doi:10.3389/fmicb.2018.01451)

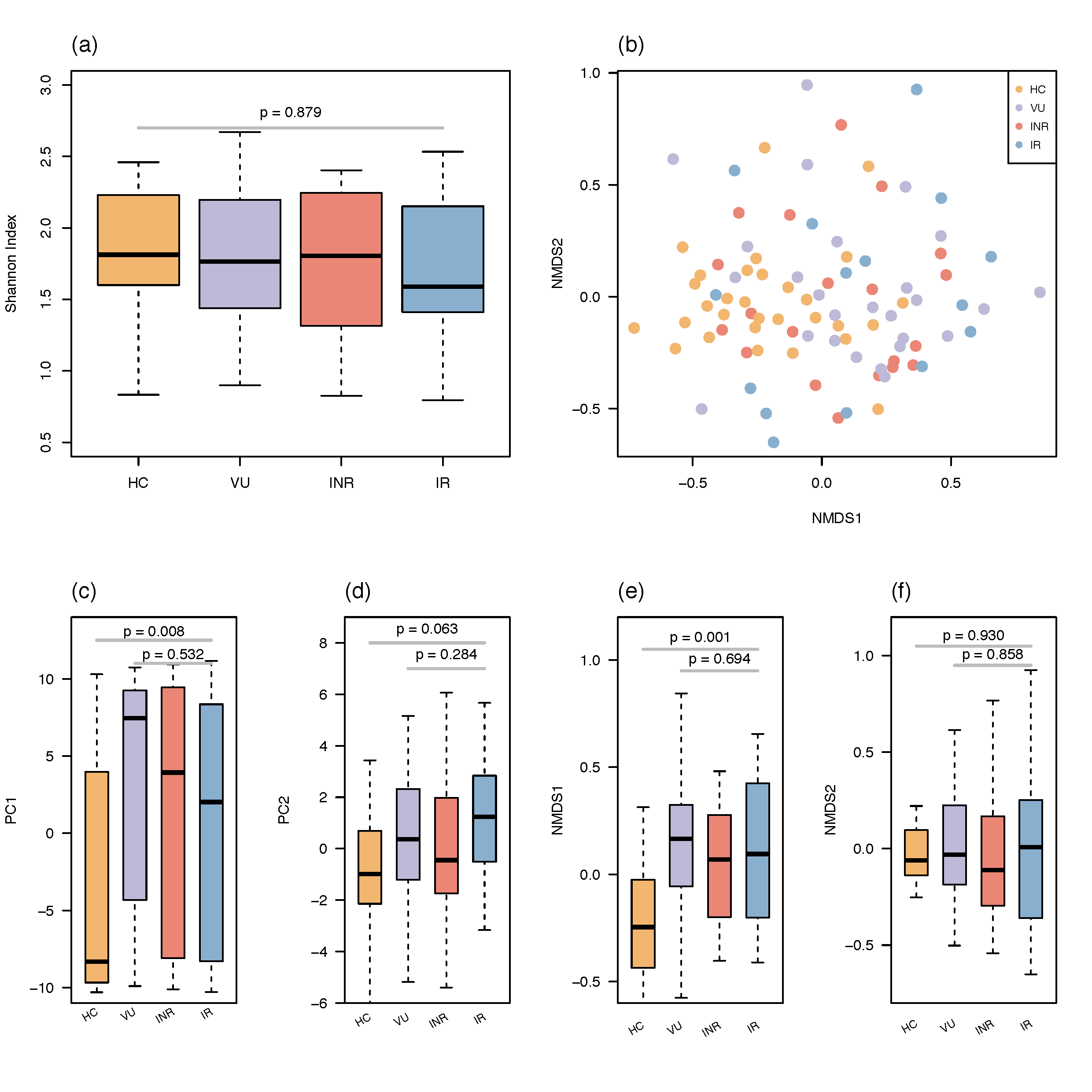

Supplement: FIGURE S1 — The differences of microbiota composition among the four groups. (A) Shannon index. (B) Non-metric multidimensional scaling (NMDS) of the bacterial composition at the genus level. (C,D) The distribution of the points along PC1 and PC2 are shown in the boxes. (E,F) The distribution of the points along NMDS1 and NMDS2 are shown in the boxes. [file Image_1.TIFF]

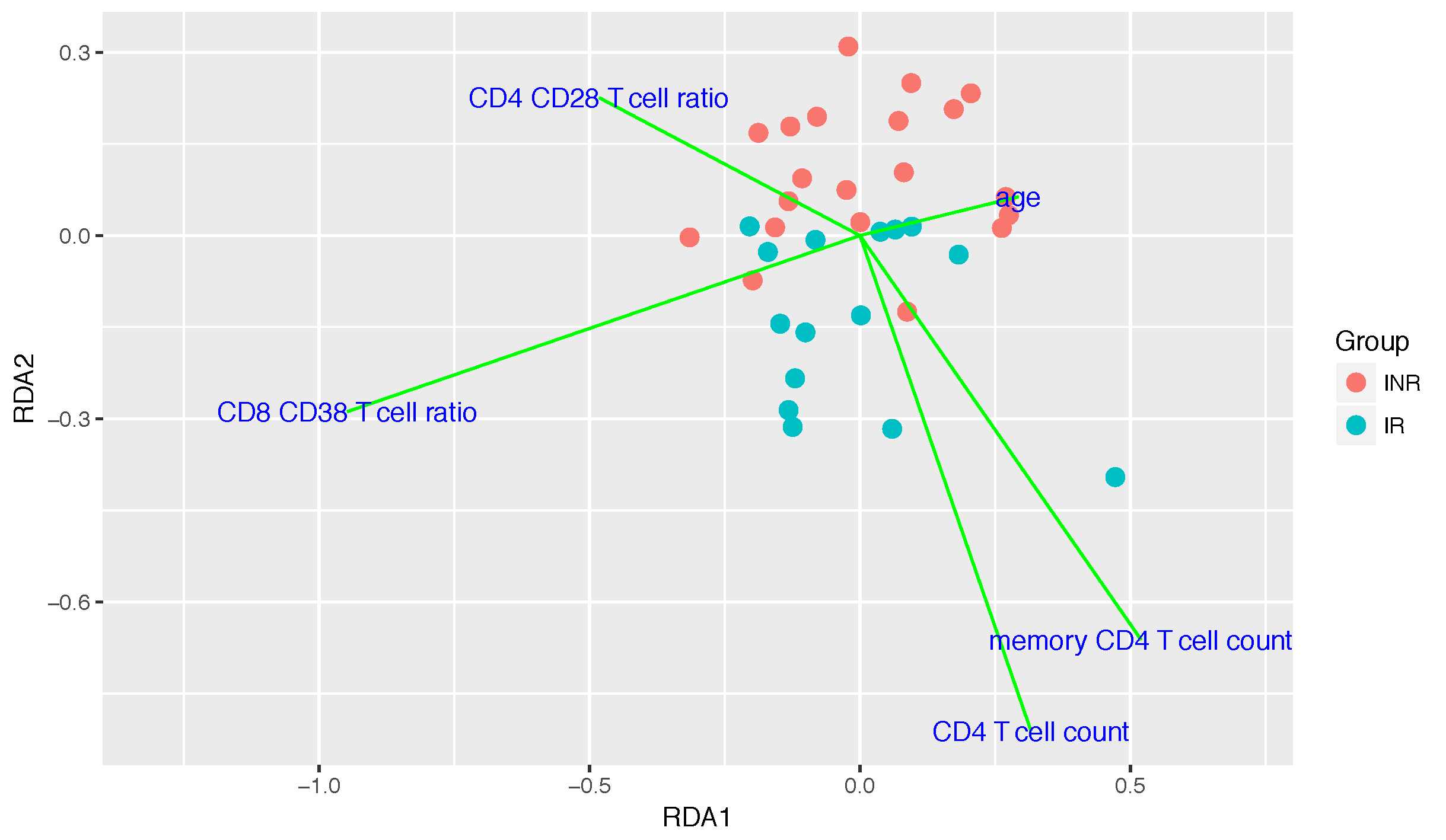

Supplement: FIGURE S2 — Redundancy Analysis (RDA) of the correlation between some clinical index and species in IR and INR groups. Red dot: patients from the INR group; blue dot: patients from the IR group; black dot: species; green line: clinical index. [file Image_2.TIFF]

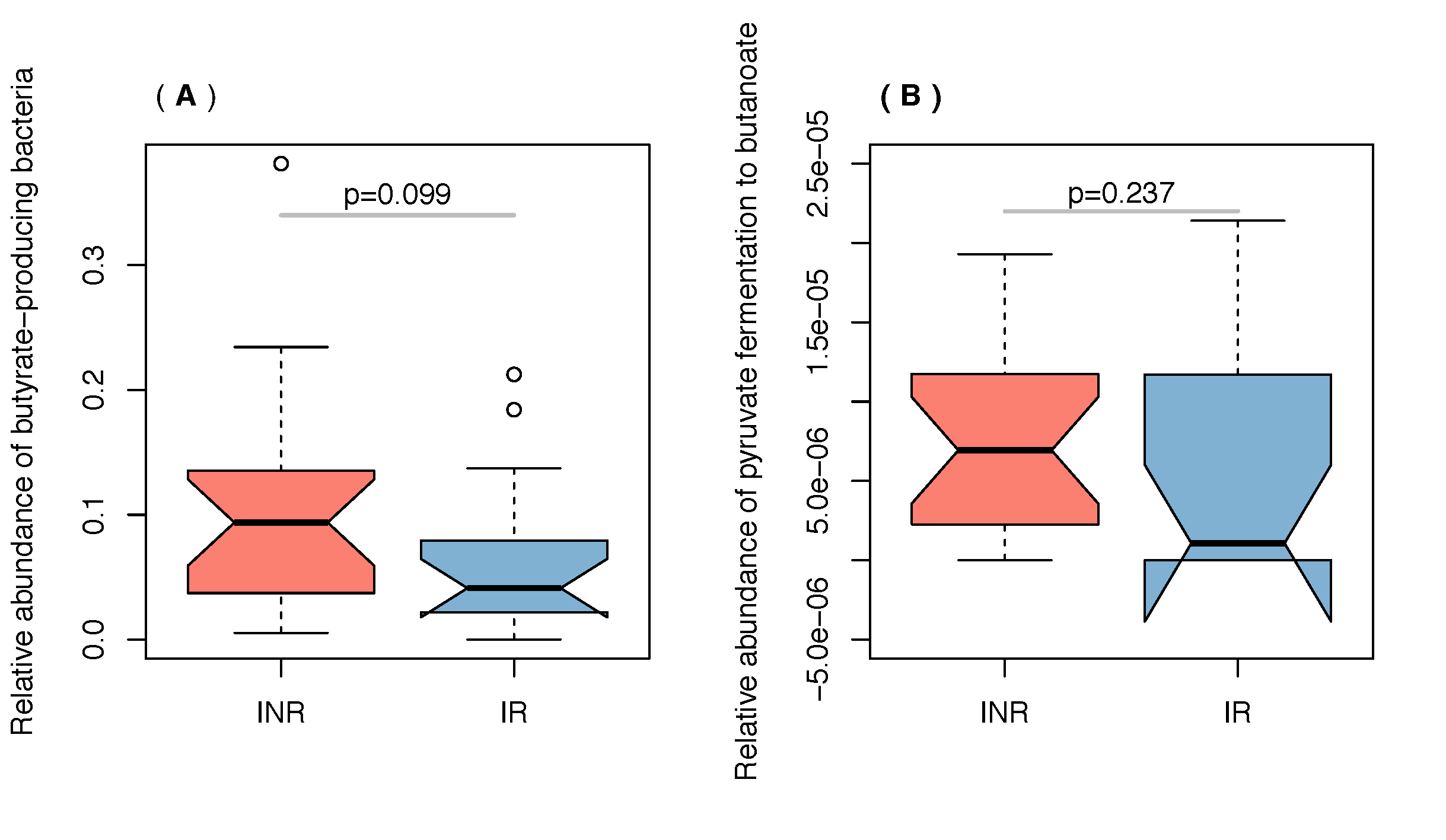

Supplement: FIGURE S3 — (A) Relative abundance of butyrate-producing bacteria. (B) Relative abundance of butyrate-producing pathway, byruvate fermentation to butanoate, acquired from HUMAnN2. [file Image_3.TIFF]

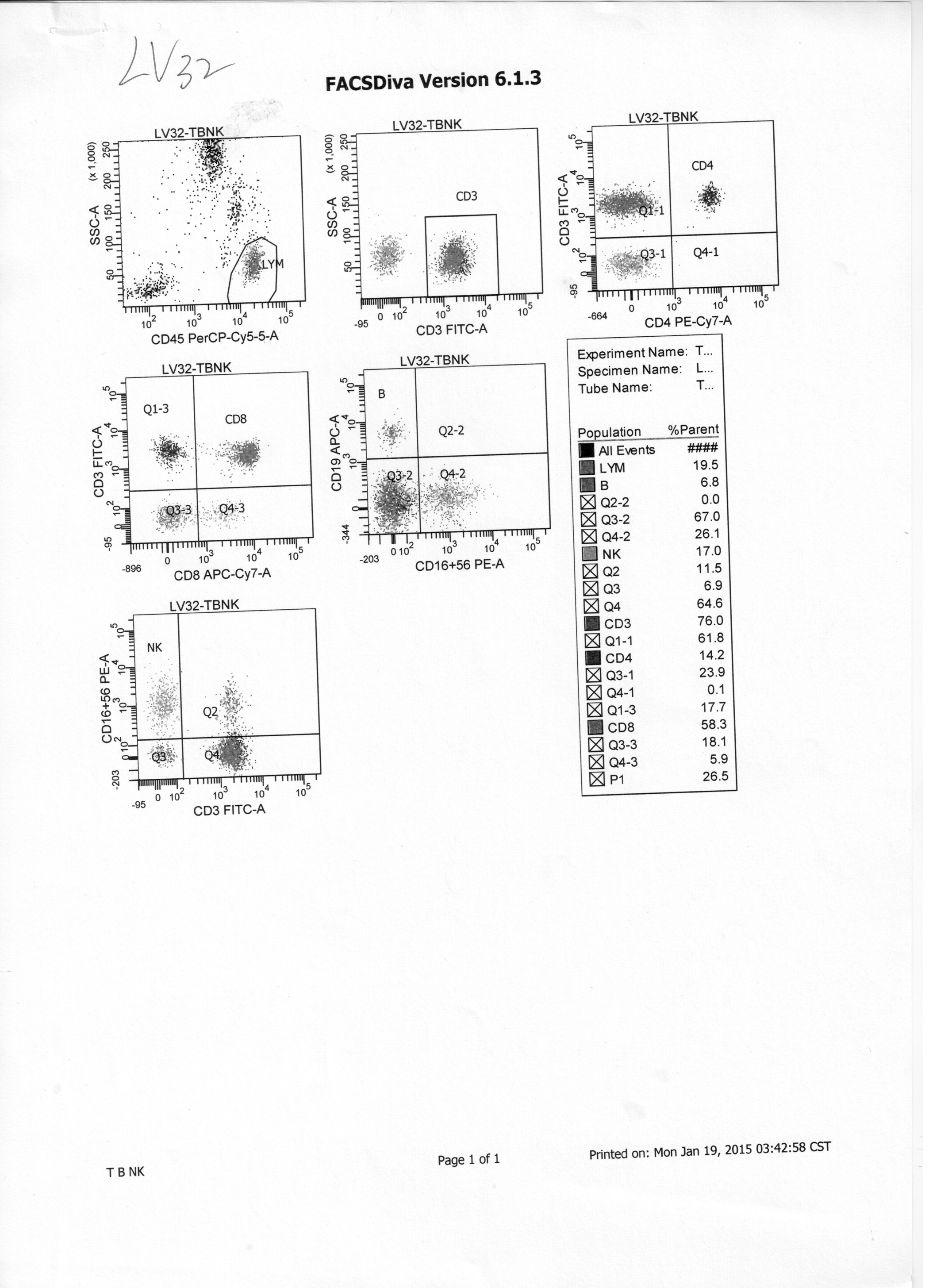

Supplement: FIGURE S4 — Flow cytometry analysis of peripheral blood in patient-1 (part 1). [file Image_4.TIF]

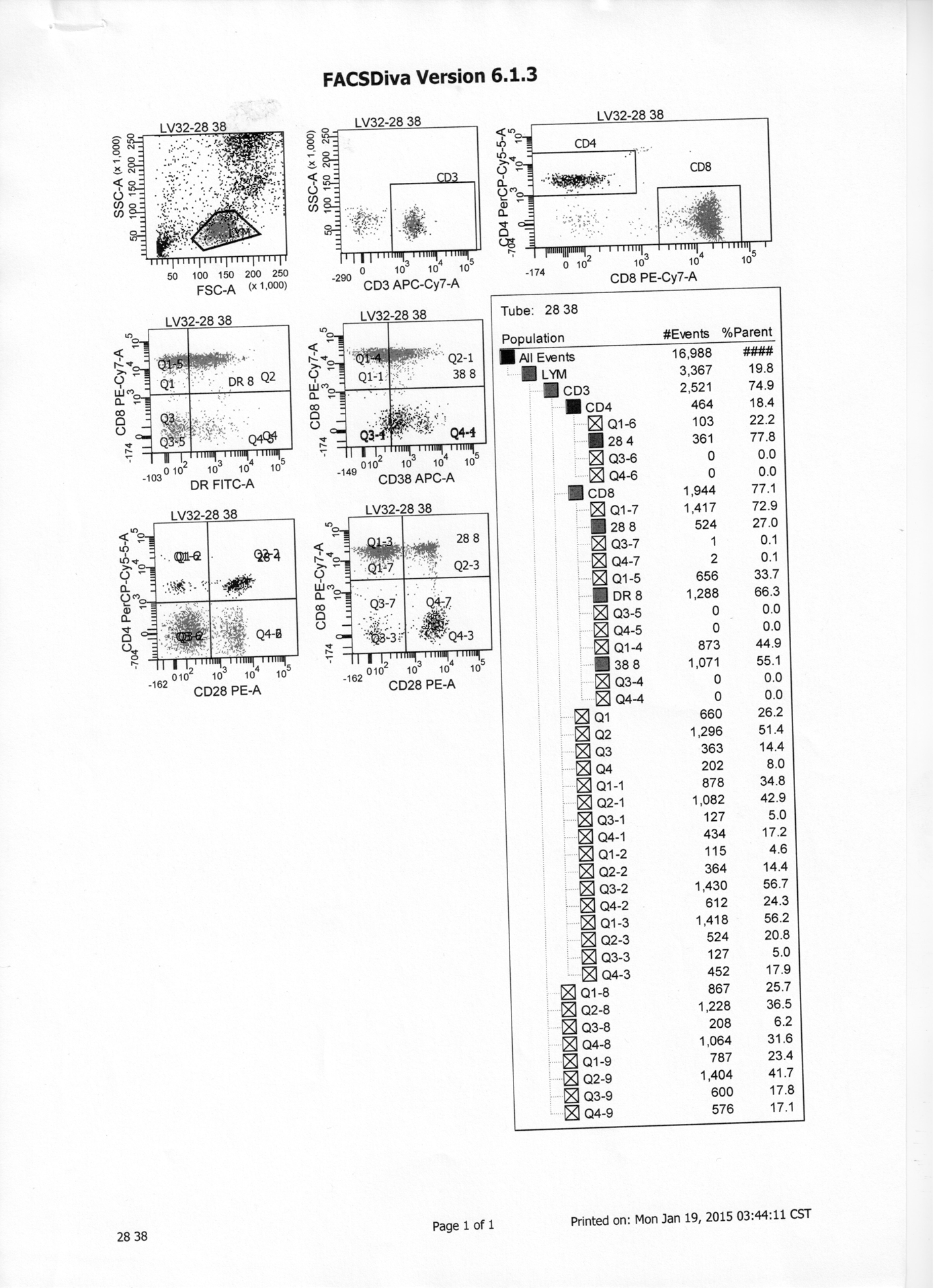

Supplement: FIGURE S5 — Flow cytometry analysis of peripheral blood in patient-1 (part 2). [file Image_5.TIF]

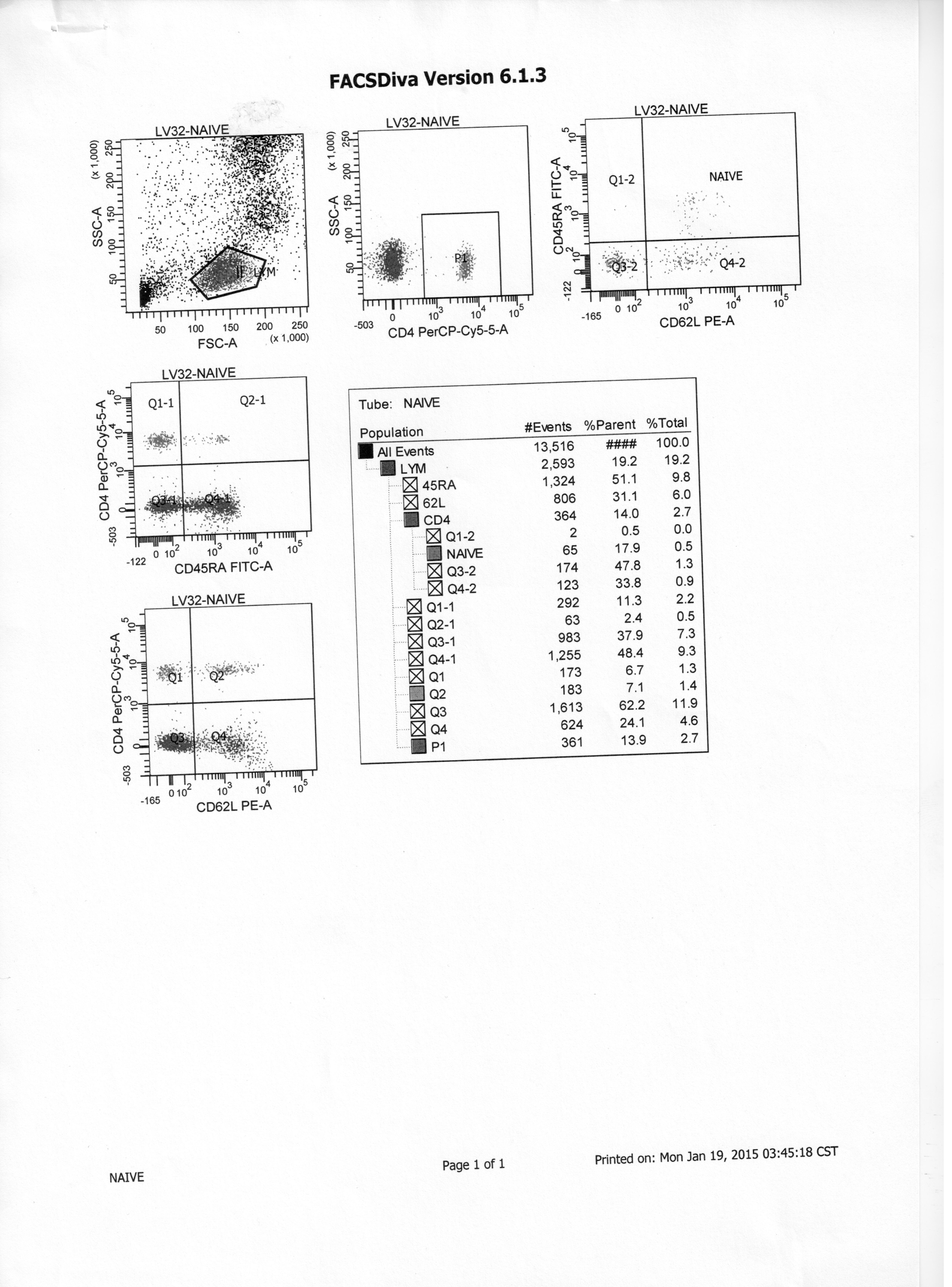

Supplement: FIGURE S6 — Flow cytometry analysis of peripheral blood in patient-1 (part 3). [file Image_6.TIF]

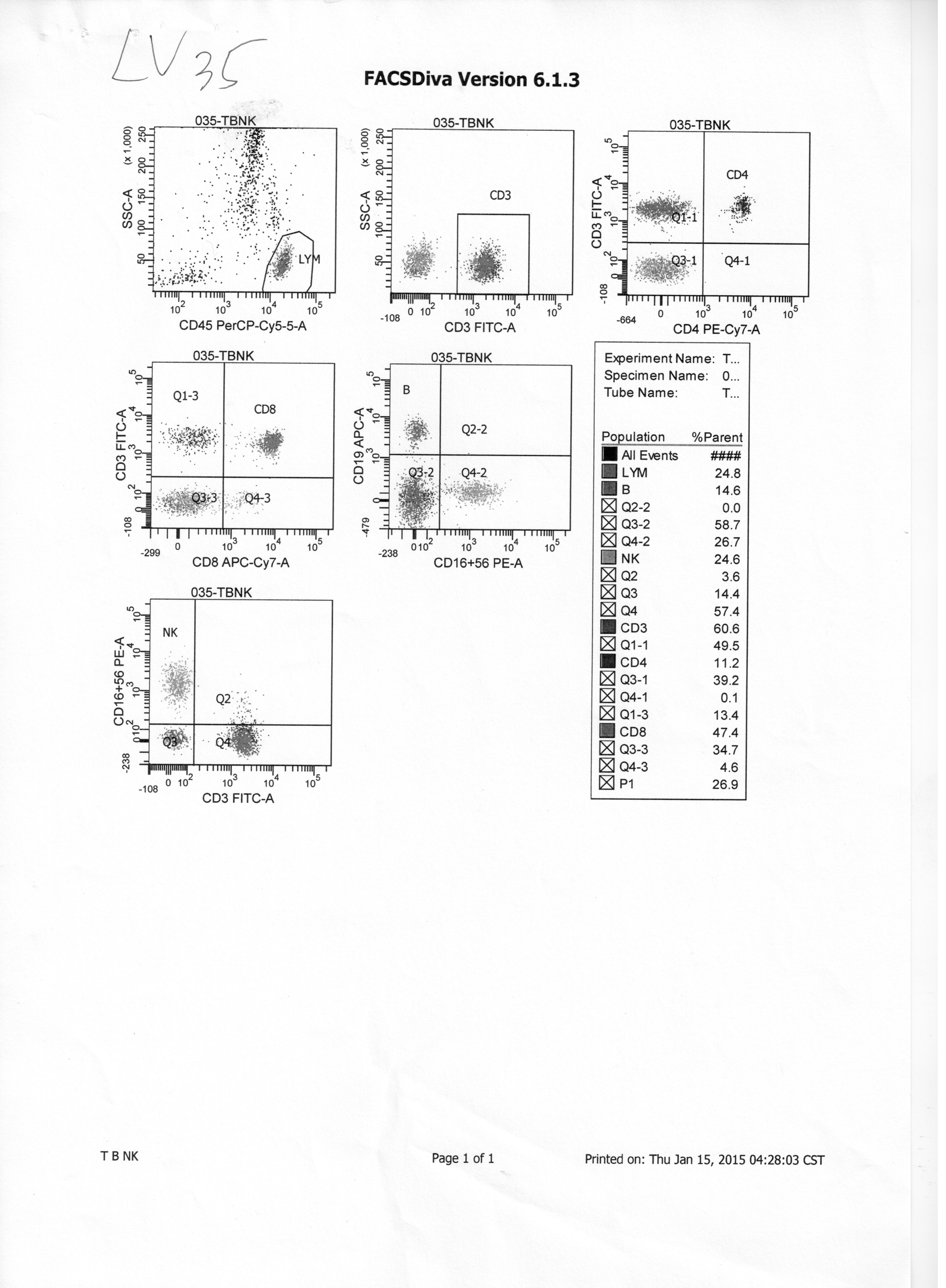

Supplement: FIGURE S7 — Flow cytometry analysis of peripheral blood in patient-2 (part 1). [file Image_7.TIF]

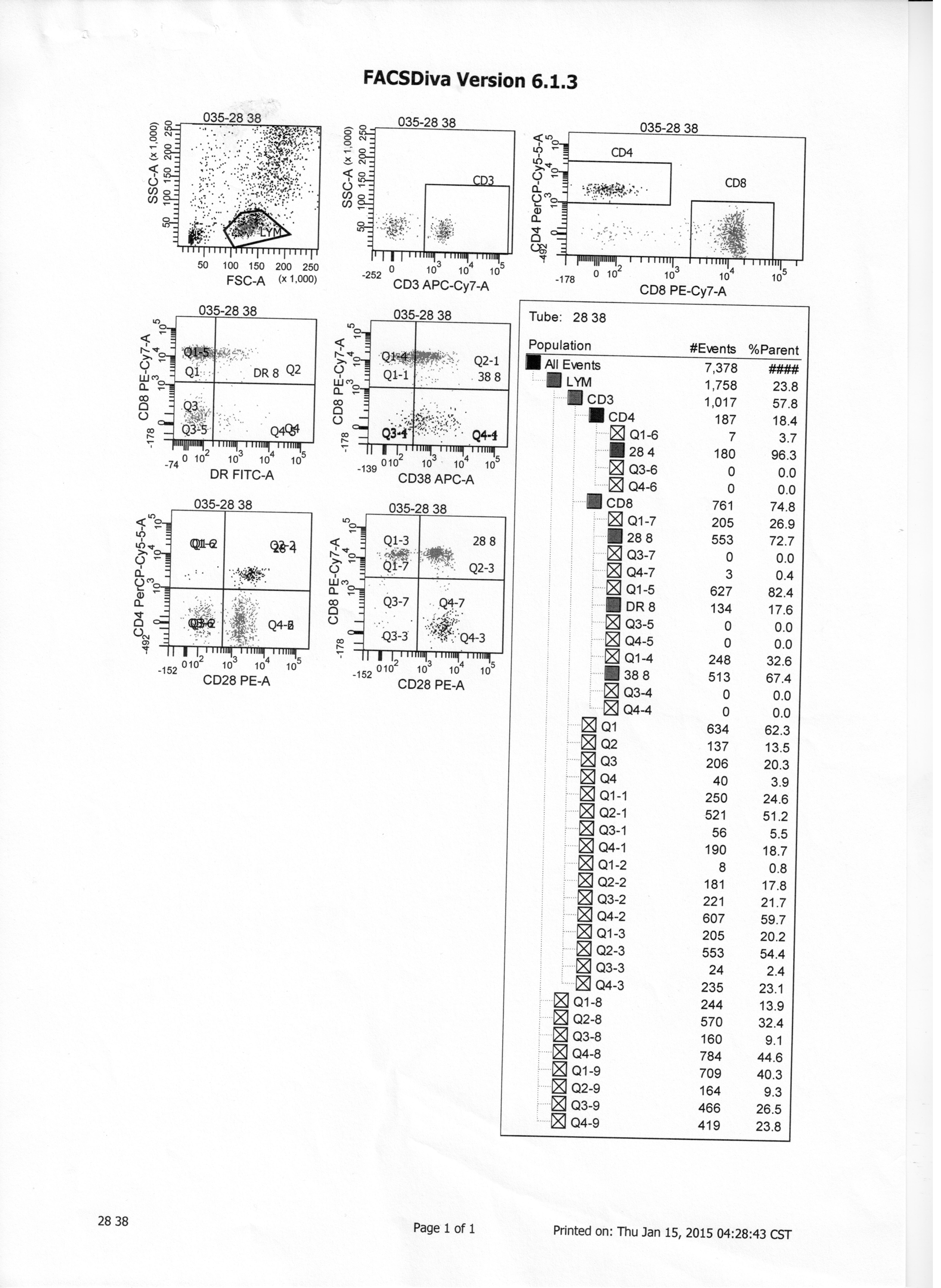

Supplement: FIGURE S8 — Flow cytometry analysis of peripheral blood in patient-2 (part 2). [file Image_8.TIF]

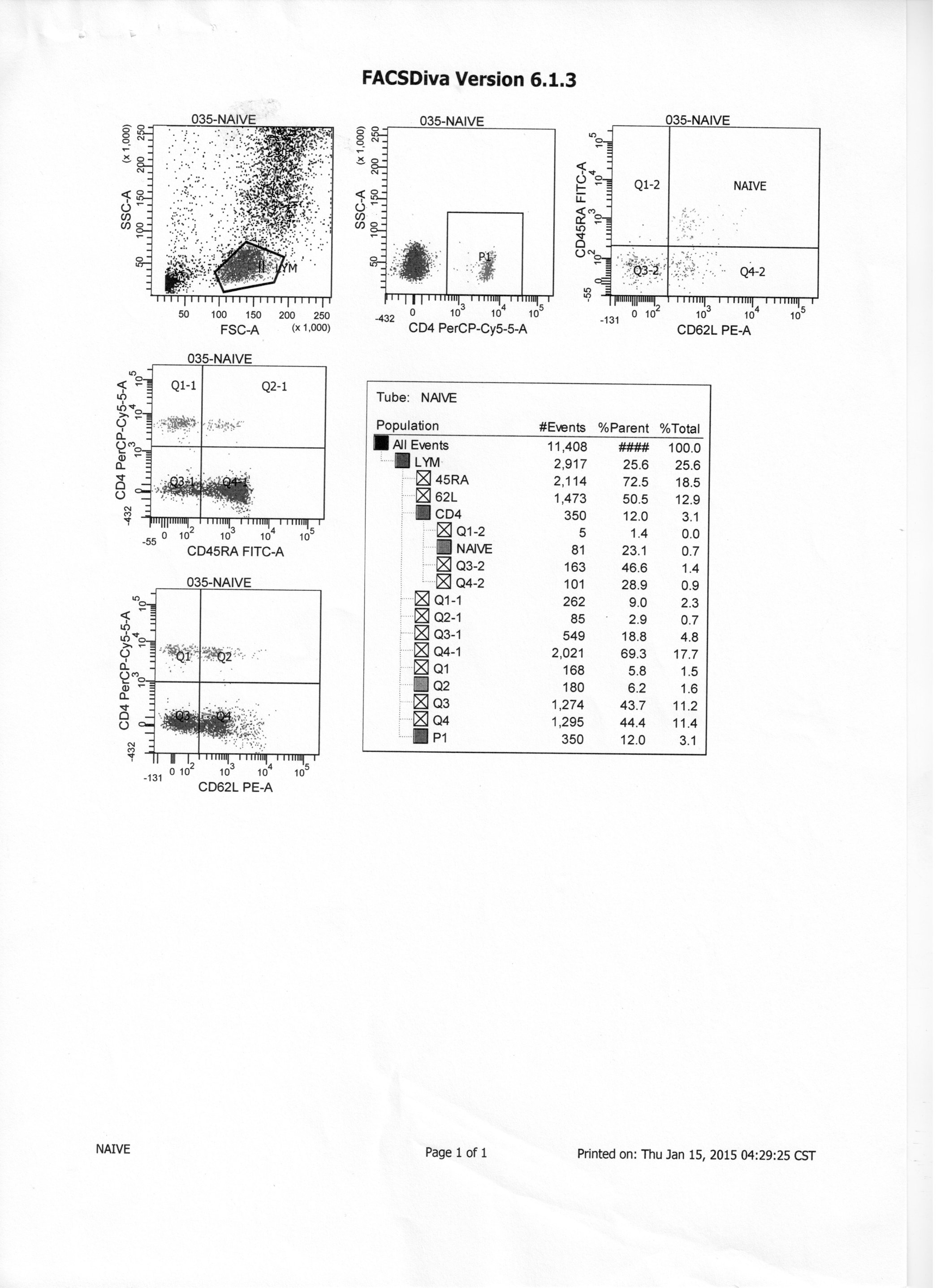

Supplement: FIGURE S9 — Flow cytometry analysis of peripheral blood in patient-2 (part 3). [file Image_9.TIF]
